# *In vivo* Emergence of Colistin and Tigecycline Resistance in Carbapenem-Resistant Hypervirulent *Klebsiella pneumoniae* During Antibiotics Treatment

**DOI:** 10.3389/fmicb.2021.702956

**Published:** 2021-09-16

**Authors:** Jiawei Chen, Yu Zeng, Rong Zhang, Jiachang Cai

**Affiliations:** Clinical Microbiology Laboratory, The Second Affiliated Hospital of Zhejiang University School of Medicine, Zhejiang University, Hangzhou, China

**Keywords:** carbapenem-resistant hypervirulent *Klebsiella pneumonia*, colistin resistance, *mgrB*, tigecycline resistance, *tet*(A)

## Abstract

Three carbapenem-resistant *Klebsiella pneumoniae* (CRKP; strains KP-426, KP-C76, and KP-CT77) were isolated from a patient with severe burns during the treatment of colistin and tigecycline. Single-nucleotide polymorphism typing showed that three ST11 CRKP were clonally related. Three isolates harbored the same set of antimicrobial resistance genes. *bla*_KPC-2_, *bla*_SHV-12_, *bla*_TEM-1_, and *rmtB* genes were located on the same 128,928-bp IncFII/IncR plasmid. *Tet*(A), *catA2*, *sul2*, and *dfrA14* genes were located on a plasmid with an unknown Inc-type. *bla*_SHV-11_, *fosA*, and *aadA2* were chromosomal genes. An IS*1* and an IS*Kpn14* were found in the promoter region of the *mgrB* gene of two colistin-resistant CRKP, *K. pneumoniae* KP-C76, and KP-CT77, respectively. A novel amino acid substitution, G300E, was identified in the type 1 Tet(A) variant of *K. pneumoniae* KP-CT77 which exhibited high-level tigecycline resistance compared to strains KP-426 and KP-C76 (MIC of 32, 4, and 4mg/l, respectively). Conjugation and cloning experiments confirmed that the mutated Tet(A) resulted in a 4-fold increase in tigecycline minimal inhibitory concentration (MIC) of *Escherichia coli*. Three CRKP belonged to the K64 serotype and possessed a similar IncHI1B/repB virulence plasmid carrying *rmpA*, *rmpA2*, and *iucABCDiutA*. The survival rates of *Galleria Mellonella* injected with *K. pneumoniae* KP-426, KP-C76, and KP-CT77 were 4.2, 20.8, and 8.3%, respectively. The emergence of colistin and tigecycline resistance in carbapenem-resistant hypervirulent *K. pneumoniae* posed a serious threat to clinical anti-infective therapy. The type 1 Tet(A) variant carrying G300E mutation, which conferred significantly elevated tigecycline MIC and was located on a conjugative plasmid, needs attention.

## Introduction

Carbapenems are important antimicrobial agents for the treatment of infections caused by multidrug-resistant Gram-negative bacteria. The emergence and spread of carbapenem-resistant Enterobacterales (CRE), especially carbapenem-resistant *Klebsiella pneumoniae* (CRKP), pose a great threat to human health (Nordmann, 2014). According to the data of the China Antimicrobial Surveillance Network, the prevalence of CRKP in Chinese tertiary hospitals sharply increased from 3.0% in 2005 to 26.3% in 2018 (Hu et al., 2019). This remarkable increasing trend may be attributed to the dissemination of sequence type (ST) 11 clone that harbored conservative mobile elements carrying *bla*_KPC-2_ or *bla*_NDM_ gene (Zhang et al., 2017; Hu et al., 2018). More worryingly, carbapenem-resistant hypervirulent *K. pneumoniae* (CR-hvKP) are increasing in recent years and their infections usually led to severe clinical outcomes and even death (Gu et al., 2018; Lan et al., 2021). Currently, the therapeutic options for CRE infections remain very limited. Polymyxins, tigecycline, and ceftazidime/avibactam are considered as last-resort antibiotics and fosfomycin and aminoglycosides are occasionally used (Sheu et al., 2019). Therefore, resistance to these antibiotics is a critical public health problem.

In this study, three clonally related CR-hvKP consecutively isolated from the same patient were investigated. Molecular analysis revealed the *in vivo* evolution of colistin and tigecycline resistance following the treatment with colistin and tigecycline.

## Materials and Methods

### The Patient and Bacterial Strains

A 39-year-old male with a burn injury (80% total burn surface area) received an emergency operation after admission to a hospital in Hangzhou, China, in 2020. Empirical antibiotic therapy with cefoperazone/sulbactam (1:1, 2g IV every 12h) was immediately initiated. A high fever (39.2°C) occurred on the 3rd day and cefoperazone/sulbactam was replaced with tigecycline (100mg IV every 12h) and teicoplanin (400mg IV once a day). The second debridement was done on the next day. A CRKP (strain KP-426) was isolated from the burn wound during the operation. On the 7th day, blood culture suggested CRKP bloodstream infection, and polymyxin B (500,000IU IV every 8h) was added. Polymyxin B was withdrawn on Day 13 due to the negative result of the subsequent blood cultures. However, CRKP did not be cleared from the patient’s burn wound and *Acinetobacter baumannii* (CRAB) emerged. On Day 23, a debridement was performed and a high fever (over 40°C) occurred on the same day. Thus, in addition to the continued use of tigecycline, polymyxin B was added to the treatment regimen until Day 32. During this period, CRKP and CRAB persisted in the patient’s burn wound and central venous catheter. After an operation on Day 36, vancomycin (2g IV every 6h) was added for anti-infection subsequently. Later on, a colistin-resistant CRKP (strain KP-C76) and a colistin- and tigecycline-resistant CRKP (strain KP-CT77) were isolated from the wound and urine samples, respectively. The anti-infective strategies were modified to ceftazidime/avibactam (2.5g IV every 8h) according to the antimicrobial susceptibility results. Both colistin-resistant CRKP were subsequently cleared; however, CRKP and CRAB could be detected occasionally from the central venous catheter and wound samples during the rest of the treatment. The patient received multiple operations, including wound debridement, closed drainage, negative-pressure wound therapy, flap repair, autologous dermal scaffold graft, and skin grafts. When he was discharged, most of the wounds healed. The routine antimicrobial susceptibility testing for CRKP isolates from multiple samples showed the same antimicrobial susceptibility profile. Therefore, only the initial CRKP (strain KP-426) was collected and used as colistin- and tigecycline-susceptible strain in this study. To investigate the evolutionary route of colistin and tigecycline resistance, three CRKP isolates of KP-426, KP-C76, and KP-CT77 were subjected to whole-genome sequencing (WGS) and further analysis. This study was approved by the Ethics Committee of the Second Affiliated Hospital of Zhejiang University School of Medicine (approval number 2020–734), and consent was given by the patient.

### Antimicrobial Susceptibility Testing

The minimal inhibitory concentrations (MICs) of 16 antimicrobial agents were determined using the broth microdilution method and interpreted according to the Clinical and Laboratory Standards Institute (CLSI) guidelines (Clinical and Laboratory Standards Institute, 2018, 2020). The susceptibility breakpoint for cefoperazone was applied for cefoperazone/sulbactam. Tigecycline susceptibility was interpreted using breakpoints for Enterobacterales recommended by United States Food and Drug Administration (≤2mg/l, susceptible; 4mg/l, intermediate; ≥8mg/l, resistant).^1^

### WGS and Genome Analysis

Three CRKP were sequenced using the Illumina NovaSeq 6,000 platform and the Nanopore PromethION 48. Hybrid assembly of short and long reads was conducted with Unicycler v.0.4.4 (Wick et al., 2017). The sequence types and antimicrobial resistance genes of three isolates were identified at the Center for Genomic Epidemiology (CGE) using MLST and ResFinder 4.1, respectively^2^ (Larsen et al., 2012; Zankari et al., 2012). The plasmids carrying *tet*(A), *bla*_KPC-2_, and virulence genes were annotated with both the RAST server^3^ and BLAST program,^4^ respectively (Overbeek et al., 2014). The plasmid types were identified by using PlasmidFinder 2.1 available at CGE (Carattoli et al., 2014). Comparison of sequences of plasmids carrying *tet*(A), *bla*_KPC-2_, and virulence genes was conducted using BRIG (v0.95), respectively (Alikhan et al., 2011). The Harvest suite was applied to run core-genome alignment and single-nucleotide polymorphism (SNP) calling (Treangen et al., 2014). The phylogenetic tree was constructed using Parsnp and was edited and visualized by iTOL (v3; Letunic and Bork, 2016).^5^ To investigate the mechanism of colistin resistance, the *mgrB* genes and their genetic context and the PmrA/PmrB and PhoP/PhoQ two-component regulatory systems among three CRKP were compared. The insertion sequences (ISs) were identified using ISfinder.^6^ Virulence genes were identified using Kleborate (v0.3.0). Capsular typing on the assembled sequences was conducted using Kaptive (v0.5.1; Wyres et al., 2016).

### Conjugation and Molecular Cloning Experiments

To assess the transferability of the *tet*(A) gene and its contribution to tetracycline and tigecycline resistance phenotype, conjugation experiments were carried out by the filter mating method. Rifampin-resistant *Escherichia coli* EC600 was used as the recipient strain. Transconjugants were selected on MacConkey agar plates containing 500mg/l rifampin and 20mg/l tetracycline. DNA fragments harboring *tet*(A) with and without mutation were amplified by PCR using primers (5′-CGGAATTCCCAGT TTGCGTGTCGTCAG-3′ and 5′-GCTCTAGACACTCCTAGGGCGCGTATAG-3′) which contained the EcoRI and XbaI restriction sites at the 5′ end of the primers. The amplified fragments were digested with EcoRI and XbaI (Thermo Fisher Scientific, Lithuania) and ligated to cloning vector pUC18 (TaKaRa, Dalian, China) digested with the same restriction enzymes. The recombinant plasmids carrying *tet*(A)/mutated *tet*(A) and their natural promoter regions were obtained and transformed to *E. coli* DH5α for further analysis.

### Virulence Testing in the *Galleria mellonella* Infection Model

A *Galleria mellonella* model was used to assess the virulence of three *K. pneumoniae* isolates as previously described (McLaughlin et al., 2014). Overnight cultures of *K. pneumoniae* were diluted in sterile phosphate-buffered saline to obtain a concentration of 10^8^CFU/ml. Wax moth larvae weighing 250mg to 300mg (Tianjin Huiyude Biotech Company, Tianjin, China) were injected with 10μl bacterial suspension and incubated for 48h at 35°C. The survival rate of *G. mellonella* was recorded at 18h, 24h, 42h, and 48h, respectively. The classic ST11 *K. pneumoniae* FJ8 without virulence plasmid and the hypervirulent *K. pneumoniae* 4 were used as the negative and positive control, respectively (Gu et al., 2018). Each isolate was tested in eight larvae and all experiments were done in triplicate. Kaplan–Meier survival curves were plotted using GraphPad Prism.

### Nucleotide Sequence Accession Numbers

The genomes of three CRKP have been deposited in GenBank under BioSample accession numbers SAMN20373013, SAMN20373014, and SAMN20375149.

## Results

### Antimicrobial Susceptibility Results

Three *K. pneumoniae* isolates exhibited almost the same susceptibility profile except for colistin and tigecycline (Table 1). These isolates were resistant to β-lactams, ciprofloxacin, amikacin, and tetracycline but were susceptible to ceftazidime/avibactam. The initial CRKP KP-426 isolated from the wound sample was susceptible to colistin (MIC of 1mg/l) and intermediate to tigecycline (MIC of 4mg/l). *K. pneumoniae* KP-C76 and KP-CT77, isolated from wound and urine samples on Day 36, respectively, were resistant to colistin with MIC of 4mg/l and 8mg/l, respectively. Moreover, *K. pneumoniae* KP-CT77 showed much higher tigecycline MIC than those of strains KP-426 and KP-C76 (32mg/lV.S. 4mg/l).

**Table 1:** Antimicrobial susceptibility results of three clinical *Klebsiella pneumoniae* isolates and their *E. coli* transconjugants and transformants.

Strain	MICs (mg/l)															
	IPM^a^	MEM	ETP	CAZ	CTX	TZP	SCF	CZA	FEP	ATM	CMZ	CIP	AK	TET	COL	TGC
*K. pneumoniae* KP-426	64	128	128	>128	>128	256/4	256/128	≤0.5/4	>128	>128	>128	>32	>128	>128	1	4
*K. pneumoniae* KP-C76	64	128	>128	>128	>128	256/4	256/128	≤0.5/4	>128	>128	>128	>32	>128	>128	4	4
*K. pneumoniae* KP-CT77	64	64	128	128	128	256/4	128/64	1/4	>128	>128	128	>32	>128	>128	8	32
*E. coli* EC600	≤0.5	≤0.5	≤0.5	≤0.5	≤0.5	≤4/4	≤1/0.5	≤0.5/4	≤0.5	≤0.5	1	≤0.25	≤4	0.5	1	0.125
*E. coli* EC600 transconjugant of KP-426	≤0.5	≤0.5	≤0.5	≤0.5	≤0.5	≤4/4	≤1/0.5	≤0.5/4	≤0.5	≤0.5	1	≤0.25	≤4	64	1	0.25
*E. coli* EC600 transconjugant of KP-C76	≤0.5	≤0.5	≤0.5	≤0.5	≤0.5	≤4/4	≤1/0.5	≤0.5/4	≤0.5	≤0.5	1	≤0.25	≤4	64	1	0.25
*E. coli* EC600 transconjugant of KP-CT77	≤0.5	≤0.5	≤0.5	≤0.5	≤0.5	≤4/4	≤1/0.5	≤0.5/4	≤0.5	≤0.5	1	≤0.25	≤4	128	1	1
*E. coli* DH5α	≤0.5	≤0.5	≤0.5	≤0.5	≤0.5	≤4/4	≤1/0.5	≤0.5/4	≤0.5	≤0.5	≤0.5	≤0.25	≤4	0.5	0.5	0.125
*E. coli* DH5α/pUC18	≤0.5	≤0.5	≤0.5	≤0.5	≤0.5	≤4/4	≤1/0.5	≤0.5/4	≤0.5	≤0.5	≤0.5	≤0.25	≤4	0.5	0.5	0.125
*E. coli* DH5α/pUC18-*tet*(A)	≤0.5	≤0.5	≤0.5	≤0.5	≤0.5	≤4/4	≤1/0.5	≤0.5/4	≤0.5	≤0.5	≤0.5	≤0.25	≤4	64	0.5	0.25
*E. coli* DH5α/pUC18-*tet*(A)-G300E	≤0.5	≤0.5	≤0.5	≤0.5	≤0.5	≤4/4	≤1/0.5	≤0.5/4	≤0.5	≤0.5	≤0.5	≤0.25	≤4	128	0.5	1

a IPM, imipenem; MEM, meropenem; ETP, ertapenem; CAZ, ceftazidime; CTX, cefotaxime; TZP, piperacillin/tazobactam; SCF, cefoperazone/sulbactam; CZA, ceftazidime/avibactam; FEP, cefepime; ATM, aztreonam; CMZ, cefmetazole; CIP, ciprofloxacin; AK, amikacin; TET, tetracycline; COL, colistin; and TGC, tigecycline. For piperacillin/tazobactam and ceftazidime/avibactam, the tazobactam and avibactam were tested at a fixed concentration of 4mg/l. For cefoperazone/sulbactam, the combination was tested with concentrations of 2:1 ratio (antibiotic: inhibitor).

### Genomic Analysis of CRKP

Genomes of three *K. pneumoniae* isolates with similar size of approximately 5.9 Mbp were obtained. Each CRKP contained several plasmids (Table 2). All three isolates belonged to ST11, which is the predominant ST of CRKP in China (Zhang et al., 2017). Pairwise SNP analysis showed that the number of SNPs between each *K. pneumoniae* isolate ranged from 11 to 14, suggesting that these strains belonged to the same clone according to the previous definition (Schurch et al., 2018). Antimicrobial resistance genes screening revealed the presence of the same set of resistance determinants, which conferred resistance to β-lactams including carbapenems (*bla*_KPC-2_, *bla*_TEM-1_, *bla*_SHV-11_, and *bla*_SHV-12_), fosfomycin (*fosA*), tetracycline [*tet*(A)], chloramphenicol (*catA2*), trimethoprim (*dfrA14*), sulfonamides (*sul2*), and aminoglycosides (*rmtB* and *aadA2*), in three CRKP.

**Table 2:** The genomes and their sizes of three CRKP.

Strain	Chromosome^a^	Plasmid 1	Plasmid 2	Plasmid 3	Plasmid 4	Plasmid 5	Plasmid 6	BioSample accession no.
*K. pneumoniae* KP-426	5,472,713bp (*bla*_SHV-11_, *fosA*, *aadA2*)	188,058bp (IncHI1B/repB; *rmpA*, *rmpA2*, *iucABCDiutA*)	128,928bp (IncFII/IncR; *bla*_KPC-2_, *bla*_SHV-12_, *bla*_TEM-1_, *rmtB*)	80,673bp [*tet*(A), *catA2*, *sul2*, *dfrA14*]	31,808bp (IncX4)	11,970bp (ColRNAI)	5,596bp	SAMN20373013
*K. pneumoniae* KP-C76	5,469,888bp (*bla*_SHV-11_, *fosA*, *aadA2*)	197,407bp (IncHI1B/repB; *rmpA*, *rmpA2*, *iucABCDiutA*)	128,928bp (IncFII/IncR; *bla*_KPC-2_, *bla*_SHV-12_, *bla*_TEM-1_, *rmtB*)	80,673bp [*tet*(A), *catA2*, *sul2*, *dfrA14*]	–	11,970bp (ColRNAI)	5,596bp	SAMN20373014
*K. pneumoniae* KP-CT77	5,470,680bp (*bla*_SHV-11_, *fosA*, *aadA2*)	186,446bp (IncHI1B/repB; *rmpA*, *rmpA2*, *iucABCDiutA*)	128,928bp (IncFII/IncR; *bla*_KPC-2_, *bla*_SHV-12_, *bla*_TEM-1_, *rmtB*)	92,645bp (ColRNAI) [*tet*(A), *catA2*, *sul2*, *dfrA14*]	31,808bp (IncX4)	–	5,596bp	SAMN20375149

a The plasmid Inc-type, antimicrobial resistance genes, and virulence genes were indicated in brackets if applicable.

Sequence analysis showed that the Tet(A) in *K. pneumoniae* KP-426 and KP-C76 with a mutation profile of I5R, V55M, I75V, T84A, S201A, F202S, and V203F belonged to the type 1 variant (Chiu et al., 2017). Interestingly, one novel amino acid substitution, G300E, was detected in the type 1 Tet(A) variant of tigecycline-resistant *K. pneumoniae* KP-CT77. Plasmids carrying *tet*(A) in strains KP-426, KP-C76, and KP-CT77 could be conjugated into *E. coli* EC600 with a similar conjugation efficiency of around 1×10^−6^. Three *E. coli* transconjugants showed high-level resistance to tetracycline (MICs of 64mg/l or 128mg/l) and various-level elevated tigecycline MIC. The tigecycline MIC of the transconjugant of *K. pneumoniae* KP-CT77 (1mg/l) increased by 8-fold, whereas that of the transconjugant of *K. pneumoniae* KP-C76 only increased by 2-fold (0.25mg/l), compared with that of the recipient (0.125mg/l; Table 1).

**Figure 1:**
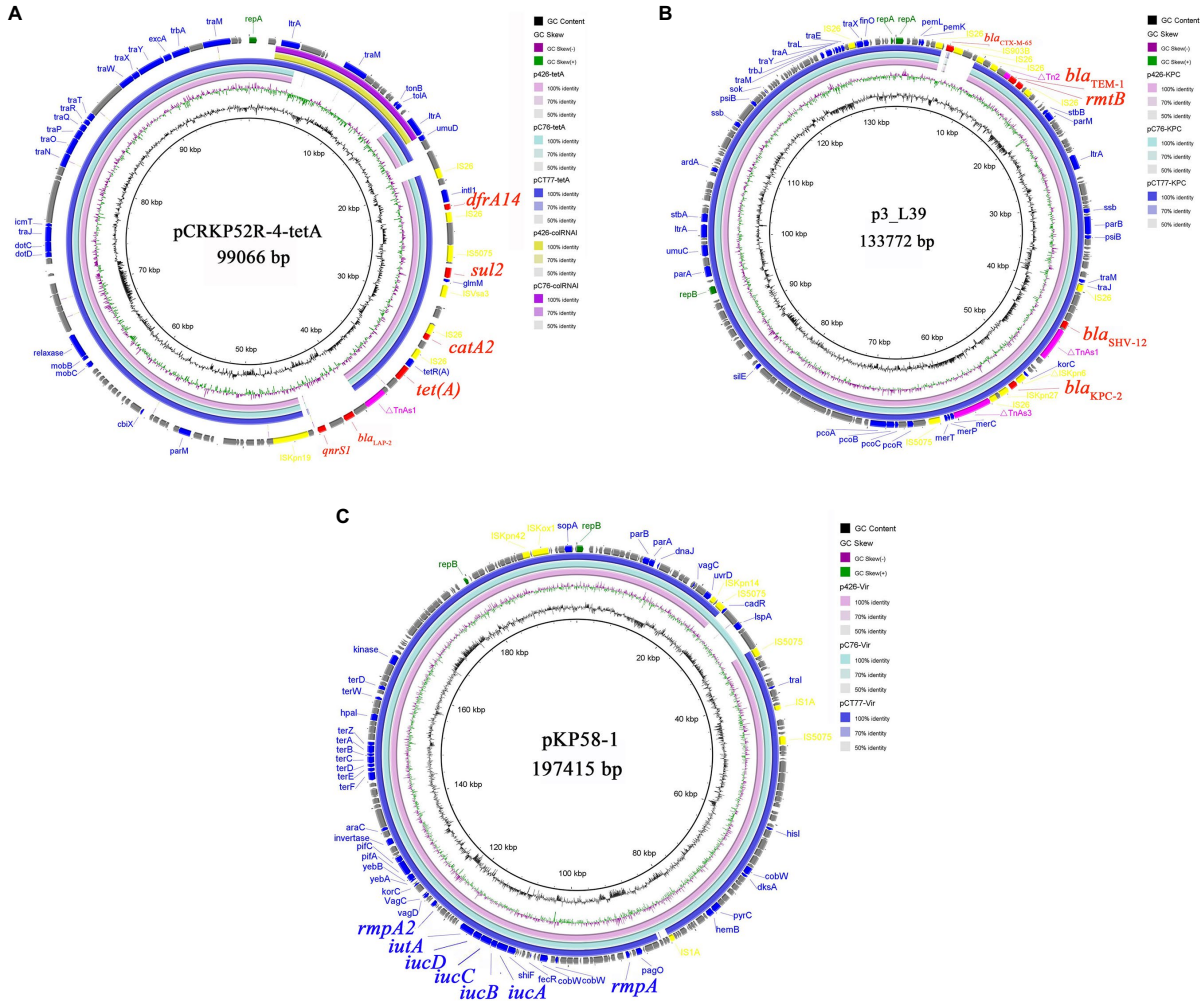
Alignment of *tet*(A)-carrying plasmids **(A)**, *bla*_KPC-2_-carrying plasmids **(B)**, and virulence plasmids **(C)** from *Klebsiella pneumoniae* KP-426, KP-C76, and KP-CT77. Circle 1 (the innermost) displayed the scale in kbp. Circle 2 and Circle 3 displayed the GC content and GC skew, respectively. The outermost circle displayed annotation of the reference plasmid.

*Klebsiella pneumoniae* KP-426 and KP-C76 harbored the same plasmid with the size of 80,673bp which co-carried *tet*(A), *catA2*, *sul2*, and *dfrA14* genes and belonged to unknown Inc-type (Figure 1A). Besides this DNA fragment, the plasmid pCT77-tetA from *K. pneumoniae* KP-CT77 contained an additional 11,972-bp DNA fragment which was similar to the sequence of an 11,972-bp ColRNAI plasmid in *K. pneumoniae* KP-426 and KP-C76 (Table 2). A similar hybrid plasmid, pCRKP52R-4-tetA (GenBank accession no. CP066252.1), which encoding the type 1 Tet(A) variant and two additional resistance determinants (QnrS1 and LAP-2), was found in a CRKP isolate from a sputum sample from a hospital in Hangzhou city. The same 128,928-bp IncFII/IncR plasmid carrying *bla*_KPC-2_, *bla*_SHV-12_, *bla*_TEM-1_, and *rmtB* genes was identified in three CRKP. The DNA sequence was extremely similar to that of a *bla*_KPC-2_-bearing plasmid p3_L39 (GenBank accession no. CP033956.1) from a fecal CRKP isolate from another hospital in Hangzhou city except for the presence of *bla*_CTX-M-65_ gene in the latter (Figure 1B).

Alignment of the *mgrB* genes and their genetic context from three CRKP revealed an insertion of a 777-bp DNA fragment in the two colistin-resistant CRKP. An IS*1* and an IS*Kpn14*, both belong to the IS*1* family, were identified between nucleotide positions −29 and −30 when referring to the *mgrB* start codon as +1, in *K. pneumoniae* KP-C76 and KP-CT77, respectively. This insertion was considered to inactivate the promoter concerning the *mgrB* expression and led to the acquired resistance to colistin (Poirel et al., 2015).

### Effects of Tet(A) and Tet(A) Carrying G300E Substitution on Tigecycline Resistance

Cloning experiments were performed to check whether the G300E substitution in the type 1 Tet(A) variant could lead to the development of tigecycline resistance. The susceptibility profile of *E. coli* DH5α carrying recombinant plasmid pUC18-*tet*(A) or pUC18-*tet*(A)-G300E was almost the same as that of transconjugants of *K. pneumoniae* KP-C76 or KP-CT77, respectively. The type 1 Tet(A) variant slightly increased the tigecycline MIC in the *E. coli* transformant (2-fold). Surprisingly, the transformant harboring the type 1 Tet(A) variant with G300E substitution showed an 8-fold increase in tigecycline MIC. These results confirmed that the G300E substitution was responsible for mediating the significantly elevated tigecycline MIC.

### Virulence Analysis

**Figure 2:**
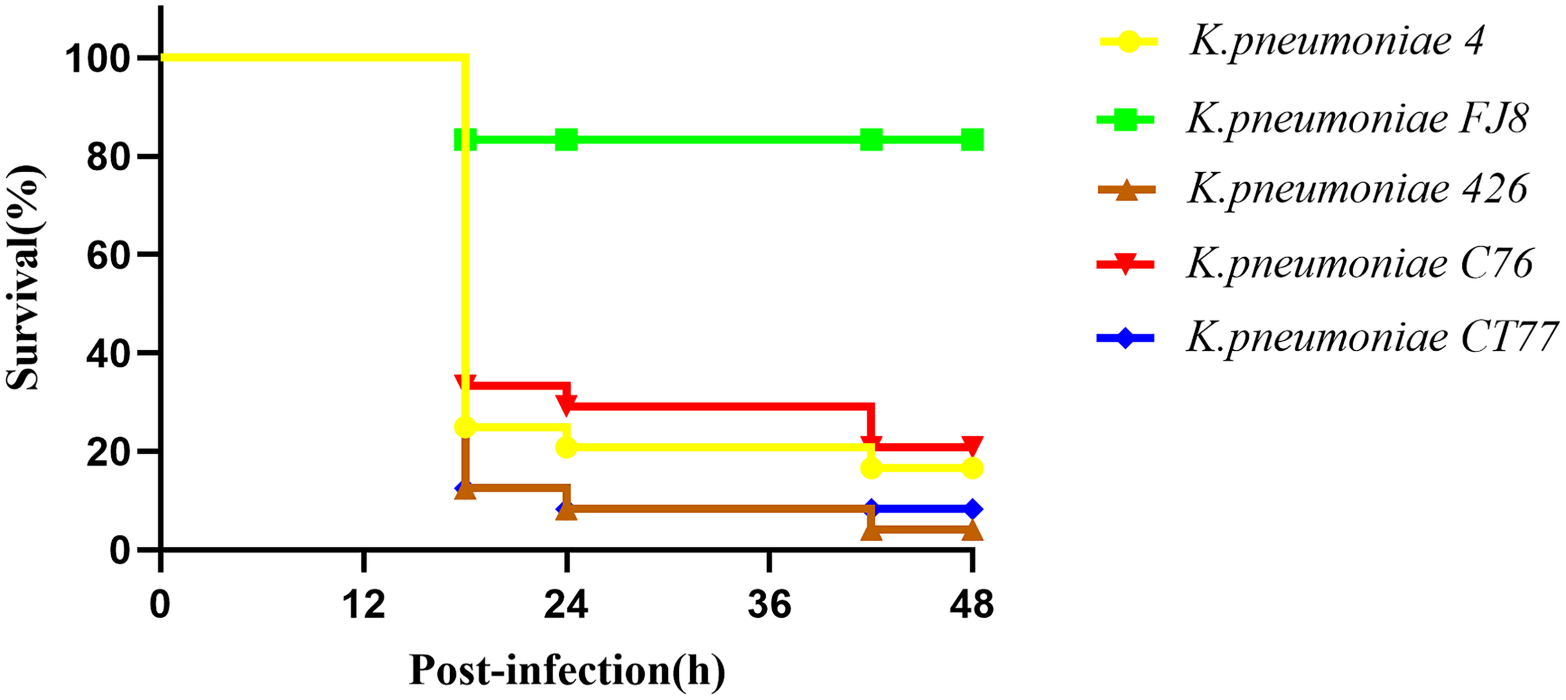
Virulence of *K. pneumoniae* isolates in the *G. mellonella* infection model.

Three CRKP belonged to the K64 serotype, which was the most common serotype among KPC-2-producing *K. pneumoniae* in China (Zhang et al., 2020). Multiple virulence-associated determinants encoding the regulators of mucoid phenotype (*rmpA* and *rmpA2* genes) and siderophores (*iucABCDiutA* cluster) were identified in an IncHI1B/repB plasmid. These virulence genes were predicted to be the best markers of hvKP (Russo and Marr, 2019), thus three CRKP were classified as CR-hvKP. Three virulence plasmids shared a similar nucleotide sequence and aligned well with the plasmid pKP58-1 (GenBank accession no. CP041374.1) which harbored in a CRKP isolate from a urine sample from a hospital in Hangzhou city (Figure 1C). The hypervirulent phenotype in three CR-hvKP was observed in the *G. mellonella* infection model. The survival rates of the larvae injected with KP-426, KP-C76, and KP-CT77 (an inoculum of 1×10^6^CFU) at 48h after infection were significantly lower than that of negative control isolate *K. pneumoniae* FJ8 (4.2, 20.8, and 8.4% V.S. 83.3%; Figure 2).

## Discussion

Colistin and tigecycline are the few therapies for serious infections caused by CRE. This study described the *in vivo* development of resistance to both antibiotics in CR-hvKP during the treatment, which greatly limited the choice of antibiotics. Ceftazidime/avibactam is the only antibiotic available in China presently that showed activity against such isolates. Worryingly, surveillance data from China showed that the *bla*_NDM_ gene, the majority of which was located on IncX3 plasmid with high conjugation efficiencies, was responsible for phenotypic resistance in 32% of the CRE (Zhang et al., 2017). What’s worse, CR-hvKP was more frequently detected than previously assumed in China (Zhang et al., 2020). These factors threatened the high risk of the acquisition of colistin, tigecycline, and ceftazidime/avibactam resistance in CR-hvKP, which would lead to serious clinical outcomes. The management and appropriate application of antibiotics are crucial to prevent this troublesome trend. Fortunately, the patient in this study has recovered after wound repair and effective anti-infection therapies.

To clear CRKP from the bloodstream and burn wound, colistin was used in combination with tigecycline for 3weeks, and two colistin-resistant CRKP were subsequently recovered from the wound and urine samples, respectively. It has been reported that alterations of the *mgrB* gene are common mechanisms of colistin resistance in *K. pneumoniae* (Cannatelli et al., 2014; Poirel et al., 2015). Interestingly, insertional inactivation of the *mgrB* gene by different insertion sequences (IS*1* and IS*Kpn14*) was identified among these two clonally related isolates, suggesting the evolution of colistin resistance in two isolates was an independent event. These findings show that prolonged use of colistin could lead to the development of colistin-resistant CRKP through IS element-mediated inactivation of the *mgrB* gene.

The type 1 Tet(A) variant was frequently detected (75%) in the tigecycline-resistant CRKP in Taiwan and was found to play an important role in tigecycline resistance (Chiu et al., 2017). In this study, *E. coli* containing the type 1 Tet(A) variant showed an only 2-fold increase in the tigecycline MIC. CRKP could not be completely cleared from the bloodstream or burn wound although tigecycline was used throughout the patient’s treatment. This phenomenon may be due to the presence of the type 1 *tet*(A) variant in these CRKP isolates (exhibiting reduced susceptibility to tigecycline in the routine antimicrobial susceptibility testing but not included in this study). Worse, a CRKP (strain KP-CT77) showed high-level tigecycline resistance mediated by a novel *tet*(A) variant carrying a G300E mutation was selected after 32days of tigecycline treatment. The G300E substitution was initially identified in the type 1 Tet(A) variant of tigecycline-resistant mutants obtained by *in vitro* selection (Linkevicius et al., 2016); however, to our best knowledge, no natural G300E mutation has been detected in clinical isolate. Plasmids analysis showed that the 92,648-bp pCT77-tetA from *K. pneumoniae* KP-CT77 was a hybrid of an 80,673-bp *tet*(A)-carrying plasmid and an 11,970-bp ColRNAI plasmid observed in both strains KP-426 and KP-C76, suggesting the evolution of *tet*(A)-carrying plasmid in *K. pneumoniae* in the presence of selective pressure *in vivo*. Since *tet*(A) and mutated *tet*(A) genes can spread through horizontal plasmid transfer, further investigations will be necessary to understand the epidemiology and evolution of *tet*(A)-carrying plasmids.

## Conclusion

We reported the *in vivo* evolution of colistin- and tigecycline-resistant CR-hvKP associated with antibiotics therapy in a Chinese patient. A novel amino acid substitution, G300E, which conferred significantly elevated tigecycline MIC, was identified in the type 1 Tet(A) variant.

## Data Availability Statement

The genomes of three *K. pneumoniae* isolates have been submitted to NCBI GenBank under BioSample accession numbers SAMN20373013, SAMN20373014, and SAMN20375149.

## Ethics Statement

The studies involving human participants were reviewed and approved by the Ethics Committee of the Second Affiliated Hospital of Zhejiang University School of Medicine. The patients/participants provided their written informed consent to participate in this study.

## Author Contributions

JCa and RZ conceived and designed the work and reviewed and improved the manuscript. JCa collected and provided the isolates. JCh, YZ, and JCa performed the experiments and analyzed the data. JCh drafted the manuscript. All authors have read and agreed to the published version of the manuscript.

## Funding

The work was supported by the National Natural Science Foundation of China (81971988) and the Zhejiang Provincial Natural Science Foundation of China (LY18H200001).

## Conflict of Interest

The authors declare that the research was conducted in the absence of any commercial or financial relationships that could be construed as a potential conflict of interest.

## Publisher’s Note

All claims expressed in this article are solely those of the authors and do not necessarily represent those of their affiliated organizations, or those of the publisher, the editors and the reviewers. Any product that may be evaluated in this article, or claim that may be made by its manufacturer, is not guaranteed or endorsed by the publisher.

## Footnotes

^1^ https://www.fda.gov/drugs/development-resources/tigecycline-injection-products

^2^ https://cge.cbs.dtu.dk/services/

^3^ http://rast.nmpdr.org/

^4^ https://blast.ncbi.nlm.nih.gov/Blast.cgi

^5^ https://itol.embl.de/

^6^ https://www-is.biotoul.fr/blast.php
